# 2-(5-Bromo-2-hydroxy­phen­yl)-1,2-dihydro­quinazolin-4(3*H*)-one

**DOI:** 10.1107/S1600536808035678

**Published:** 2008-12-24

**Authors:** Davar M. Boghaei, Mohammad Mahdi Najafpour, Vickie McKee

**Affiliations:** a Department of Chemistry, Sharif University of Technology, PO Box 11155-8639, Tehran, Iran; b Department of Chemistry, Loughborough University, Leicestershire LE11 3TU, England

## Abstract

The asymmetric unit of the title compound, C_14_H_11_BrN_2_O_2_, contains two independent mol­ecules connected into a dimer by inter­molecular N—H⋯O hydrogen bonds involving the amine and carbonyl groups. The dimers are further connected by O—H⋯O hydrogen bonds, forming chains running parallel to the *a* axis, which are stabilized through π–π stacking inter­actions, with a centroid–centroid distance of 3.679 (8) Å. The dihedral angle between the two aromatic rings is 89.2 (4)°.

## Related literature

For general background to the chemistry of quinazolinone derivatives, see: Liu (2008 ▶); Goto *et al.* (1993 ▶); Mohri (2001 ▶). For hydrogen-bond motifs, see: Bernstein *et al.* (1995 ▶).
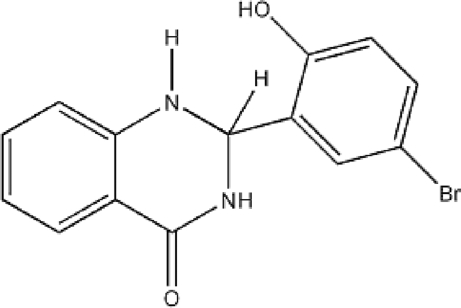


## Experimental

### 

#### Crystal data

C_14_H_11_BrN_2_O_2_
*M* *_r_* = 319.16
Triclinic, 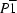

*a* = 8.8392 (5) Å
*b* = 11.2252 (7) Å
*c* = 13.8817 (8) Å
α = 73.0392 (9)°
β = 75.9620 (9)°
γ = 85.0936 (9)°
*V* = 1277.95 (13) Å^3^
*Z* = 4
Mo *K*α radiation
μ = 3.21 mm^−1^
*T* = 150 (2) K
0.21 × 0.12 × 0.07 mm

#### Data collection

Bruker APEXII CCD diffractometer
Absorption correction: multi-scan (*SADABS*; Sheldrick, 2003 ▶) *T*_min_ = 0.552, *T*_max_ = 0.806
13015 measured reflections
6129 independent reflections
4580 reflections with *I* > 2σ(*I*)
*R*_int_ = 0.035

#### Refinement

*R*[*F* ^2^ > 2σ(*F* ^2^)] = 0.040
*wR*(*F* ^2^) = 0.099
*S* = 1.02
6129 reflections
343 parameters
H-atom parameters constrained
Δρ_max_ = 0.64 e Å^−3^
Δρ_min_ = −0.70 e Å^−3^

### 

Data collection: *APEX2* (Bruker, 2005 ▶); cell refinement: *SAINT* (Bruker, 2005 ▶); data reduction: *SAINT*; program(s) used to solve structure: *SHELXS97* (Sheldrick, 2008 ▶); program(s) used to refine structure: *SHELXL97* (Sheldrick, 2008 ▶); molecular graphics: *SHELXTL* (Sheldrick, 2008 ▶); software used to prepare material for publication: *SHELXTL*.

## Supplementary Material

Crystal structure: contains datablocks I, global, New_Global_Publ_Block. DOI: 10.1107/S1600536808035678/rz2254sup1.cif

Structure factors: contains datablocks I. DOI: 10.1107/S1600536808035678/rz2254Isup2.hkl

Additional supplementary materials:  crystallographic information; 3D view; checkCIF report

## Figures and Tables

**Table 1:** Hydrogen-bond geometry (Å, °)

*D*—H⋯*A*	*D*—H	H⋯*A*	*D*⋯*A*	*D*—H⋯*A*
N2*A*—H2*NA*⋯O1*B*	0.95	1.97	2.897 (3)	165
N2*B*—H2*NB*⋯O1*A*	0.91	2.05	2.914 (3)	157
O2*A*—H2*OA*⋯O1*A*^i^	0.85	1.90	2.701 (3)	157
O2*B*—H2*OB*⋯O1*B*^ii^	0.85	1.86	2.691 (3)	165

Symmetry codes: (i) 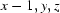; (ii) 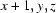.

**Fig. 1.**
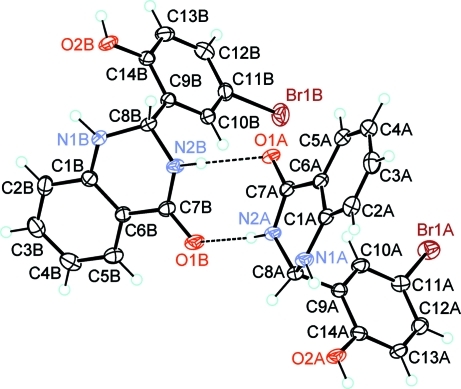
The molecular structure of the title compound with displacement ellipsoids drawn at the 50% probability level. Hydrogen bonds are shown as dashed lines.

**Fig. 2.**
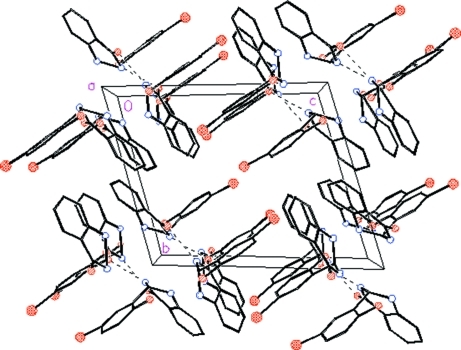
Crystal packing of the title compound viewed along the a axis with hydrogen atoms omitted for clarity. Hydrogen bonds are shown as dashed lines.

## supplementary crystallographic information

### Comment

Quinazolinone derivatives are of interest because of their biological activity, and have been widely used as key compounds in medicinal drugs (Goto *et al.*, 1993; Mohri, 2001). We herein report the crystal structure of 1,2-dihydro-2-(5-bromo-2-hydroxybenzene)-4(3*H*)-quinazolinone.

The asymmentric unit of the title compound (Fig. 1) contains two crystallographically independent molecules, which are linked into a dimer by a pair of intermolecular N—H···O hydrogen bonds (Table 1), generating a ring of graph set *R*^2^_2_(8) (Bernstein *et al.*, 1995). Bond lengths and angles are within normal ranges. The dimers are further connected by O—H···O hydrogen bonding interactions to form chains running parallel to the *a* axis (Figures 2). The chains are stabilized by π–π stacking interactions involving adjacent 5-bromo-2-hydroxybenzene rings, with a centroid-centroid separation of 3.679 (8) Å, a perpendicular interplanar distance of 3.561 (8) Å and a centroid···centroid offset of 0.924 (6) Å.

### Experimental

The title compound was synthesized by adding 5-bromo-2-hydroxybenzaldehyde (2 mmol, 402 mg) to a solution of 2-aminobenzamide (2 mmol, 272 mg) and manganese acetate (0.02 mmol, 4.90 mg) in ethanol (20 ml). The mixture was refluxed with stirring for 5 h. The resultant yellow solution was filtered. Yellow single crystals of the title compound suitable for *X*-ray structure determination were recrystallized from a mixture of water/ethanol (2:1 v/v) by slow evaporation of the solvents at room temperature over several days.

### Refinement

All H atoms atoms were placed in calculated positions and refined using the riding model approximation, with C—H = 0.95-1.0 Å, O—H = 0.85 Å, N—H = 0.89 Å and with *U*_iso_(H) = 1.2*U*_eq_(C). The isotropic thermal parameter of the hydrogen atoms bound to the N and O atoms was fixed at 0.04 Å^2^.

### Crystal data

**Table:**

C_14_H_11_BrN_2_O_2_	*Z* = 4
*M**_r_* = 319.16	*F*(000) = 640
Triclinic, *P*1	*D*_x_ = 1.659 Mg m^−^^3^
Hall symbol: -P 1	Mo *K*α radiation, λ = 0.71073 Å
*a* = 8.8392 (5) Å	Cell parameters from 3058 reflections
*b* = 11.2252 (7) Å	θ = 2.4–25.8°
*c* = 13.8817 (8) Å	µ = 3.22 mm^−^^1^
α = 73.0392 (9)°	*T* = 150 K
β = 75.9620 (9)°	Block, yellow
γ = 85.0936 (9)°	0.21 × 0.12 × 0.07 mm
*V* = 1277.95 (13) Å^3^	

### Data collection

**Table:**

Bruker APEXII CCD diffractometer	6129 independent reflections
Radiation source: fine-focus sealed tube	4580 reflections with *I* > 2σ(*I*)
graphite	*R*_int_ = 0.035
φ and ω scans	θ_max_ = 28.0°, θ_min_ = 1.6°
Absorption correction: multi-scan (*SADABS*; Sheldrick, 2003)	*h* = −11→11
*T*_min_ = 0.552, *T*_max_ = 0.806	*k* = −14→14
13015 measured reflections	*l* = −18→18

### Refinement

**Table:**

Refinement on *F*^2^	Primary atom site location: structure-invariant direct methods
Least-squares matrix: full	Secondary atom site location: difference Fourier map
*R*[*F*^2^ > 2σ(*F*^2^)] = 0.040	Hydrogen site location: inferred from neighbouring sites
*wR*(*F*^2^) = 0.099	H-atom parameters constrained
*S* = 1.02	*w* = 1/[σ^2^(*F*_o_^2^) + (0.0415*P*)^2^ + 0.6583*P*] where *P* = (*F*_o_^2^ + 2*F*_c_^2^)/3
6129 reflections	(Δ/σ)_max_ = 0.001
343 parameters	Δρ_max_ = 0.64 e Å^−^^3^
0 restraints	Δρ_min_ = −0.70 e Å^−^^3^

### Special details

**Table:**

Geometry. All e.s.d.'s (except the e.s.d. in the dihedral angle between two l.s. planes) are estimated using the full covariance matrix. The cell e.s.d.'s are taken into account individually in the estimation of e.s.d.'s in distances, angles and torsion angles; correlations between e.s.d.'s in cell parameters are only used when they are defined by crystal symmetry. An approximate (isotropic) treatment of cell e.s.d.'s is used for estimating e.s.d.'s involving l.s. planes.
Refinement. Refinement of *F*^2^ against all reflections. The weighted *R*-factor *wR* and goodness of fit *S* are based on *F*^2^, conventional *R*-factors *R* are based on *F*, with *F* set to zero for negative *F*^2^. The threshold expression of *F*^2^ > σ(*F*^2^) is used only for calculating *R*-factors(gt) *etc*. and is not relevant to the choice of reflections for refinement. *R*-factors based on *F*^2^ are statistically about twice as large as those based on *F*, and *R*- factors based on all data will be even larger.

### Fractional atomic coordinates and isotropic or equivalent isotropic displacement parameters (Å^2^)

**Table:**

	*x*	*y*	*z*	*U*_iso_*/*U*_eq_	
N1A	0.0154 (3)	−0.1619 (2)	0.82388 (19)	0.0233 (5)	
C1A	0.1438 (3)	−0.2154 (3)	0.7715 (2)	0.0226 (6)	
C2A	0.1414 (4)	−0.3350 (3)	0.7594 (2)	0.0274 (7)	
H2A	0.0487	−0.3822	0.7874	0.033*	
C3A	0.2724 (4)	−0.3839 (3)	0.7072 (2)	0.0308 (7)	
H3A	0.2693	−0.4652	0.7000	0.037*	
C4A	0.4098 (4)	−0.3167 (3)	0.6646 (2)	0.0301 (7)	
H4A	0.4981	−0.3506	0.6262	0.036*	
C5A	0.4164 (3)	−0.2011 (3)	0.6785 (2)	0.0260 (7)	
H5A	0.5103	−0.1554	0.6508	0.031*	
C6A	0.2848 (3)	−0.1506 (3)	0.7335 (2)	0.0221 (6)	
C7A	0.2963 (3)	−0.0355 (3)	0.7617 (2)	0.0219 (6)	
O1A	0.4227 (2)	0.0103 (2)	0.75507 (16)	0.0260 (5)	
N2A	0.1608 (3)	0.0141 (2)	0.80201 (19)	0.0227 (5)	
C8A	0.0102 (3)	−0.0267 (3)	0.7988 (2)	0.0224 (6)	
H8A	−0.0707	−0.0030	0.8545	0.027*	
C9A	−0.0345 (3)	0.0361 (3)	0.6958 (2)	0.0198 (6)	
C10A	0.0667 (3)	0.1068 (3)	0.6088 (2)	0.0222 (6)	
H10A	0.1705	0.1192	0.6113	0.027*	
C11A	0.0160 (3)	0.1594 (3)	0.5183 (2)	0.0232 (6)	
Br1A	0.15542 (4)	0.25729 (3)	0.40004 (2)	0.03233 (10)	
C12A	−0.1353 (3)	0.1454 (3)	0.5130 (2)	0.0246 (6)	
H12A	−0.1687	0.1827	0.4507	0.029*	
C13A	−0.2376 (3)	0.0763 (3)	0.5999 (2)	0.0248 (6)	
H13A	−0.3421	0.0664	0.5973	0.030*	
C14A	−0.1883 (3)	0.0214 (3)	0.6905 (2)	0.0212 (6)	
O2A	−0.2832 (2)	−0.0477 (2)	0.77902 (16)	0.0265 (5)	
N1B	0.5589 (3)	0.1904 (2)	1.02282 (18)	0.0235 (5)	
C1B	0.4498 (3)	0.2837 (3)	1.0340 (2)	0.0215 (6)	
C2B	0.4523 (4)	0.3566 (3)	1.1007 (2)	0.0272 (7)	
H2B	0.5320	0.3436	1.1381	0.033*	
C3B	0.3386 (4)	0.4471 (3)	1.1116 (2)	0.0297 (7)	
H3B	0.3396	0.4949	1.1577	0.036*	
C4B	0.2227 (4)	0.4691 (3)	1.0563 (2)	0.0306 (7)	
H4B	0.1478	0.5341	1.0620	0.037*	
C5B	0.2169 (3)	0.3961 (3)	0.9929 (2)	0.0266 (7)	
H5B	0.1362	0.4095	0.9564	0.032*	
C6B	0.3289 (3)	0.3025 (3)	0.9820 (2)	0.0209 (6)	
C7B	0.3173 (3)	0.2169 (3)	0.9224 (2)	0.0224 (6)	
O1B	0.1980 (2)	0.2107 (2)	0.89065 (17)	0.0307 (5)	
N2B	0.4387 (3)	0.1394 (2)	0.90652 (19)	0.0224 (5)	
C8B	0.5894 (3)	0.1544 (3)	0.9271 (2)	0.0217 (6)	
H8B	0.6427	0.0710	0.9396	0.026*	
C9B	0.6953 (3)	0.2433 (3)	0.8361 (2)	0.0203 (6)	
C10B	0.6512 (3)	0.3015 (3)	0.7437 (2)	0.0217 (6)	
H10B	0.5498	0.2894	0.7368	0.026*	
C11B	0.7546 (3)	0.3770 (3)	0.6619 (2)	0.0246 (6)	
Br1B	0.69147 (4)	0.45082 (3)	0.53632 (2)	0.03648 (11)	
C12B	0.9020 (4)	0.3972 (3)	0.6699 (2)	0.0283 (7)	
H12B	0.9714	0.4501	0.6133	0.034*	
C13B	0.9479 (3)	0.3395 (3)	0.7613 (2)	0.0258 (6)	
H13B	1.0494	0.3527	0.7675	0.031*	
C14B	0.8457 (3)	0.2624 (3)	0.8440 (2)	0.0209 (6)	
O2B	0.8846 (2)	0.2024 (2)	0.93607 (16)	0.0274 (5)	
H1NA	−0.0747	−0.1968	0.8334	0.040*	
H1NB	0.6366	0.1868	1.0581	0.040*	
H2NA	0.1709	0.0883	0.8199	0.040*	
H2NB	0.4285	0.0809	0.8750	0.040*	
H2OA	−0.3770	−0.0516	0.7751	0.040*	
H2OB	0.9813	0.2141	0.9279	0.040*	

### Atomic displacement parameters (Å^2^)

**Table:**

	*U*^11^	*U*^22^	*U*^33^	*U*^12^	*U*^13^	*U*^23^
N1A	0.0159 (11)	0.0269 (14)	0.0256 (13)	−0.0034 (10)	−0.0037 (10)	−0.0049 (11)
C1A	0.0204 (14)	0.0291 (16)	0.0183 (14)	0.0018 (12)	−0.0080 (11)	−0.0041 (12)
C2A	0.0309 (17)	0.0272 (17)	0.0253 (16)	−0.0028 (13)	−0.0102 (13)	−0.0054 (13)
C3A	0.0415 (19)	0.0265 (17)	0.0278 (16)	0.0022 (14)	−0.0123 (14)	−0.0098 (14)
C4A	0.0276 (16)	0.0382 (19)	0.0293 (17)	0.0080 (14)	−0.0100 (13)	−0.0163 (15)
C5A	0.0224 (15)	0.0331 (17)	0.0255 (15)	0.0019 (13)	−0.0102 (12)	−0.0094 (13)
C6A	0.0190 (14)	0.0276 (16)	0.0203 (14)	0.0012 (12)	−0.0084 (11)	−0.0049 (12)
C7A	0.0182 (14)	0.0290 (16)	0.0192 (14)	0.0000 (12)	−0.0076 (11)	−0.0052 (12)
O1A	0.0148 (10)	0.0353 (12)	0.0316 (12)	−0.0002 (9)	−0.0082 (9)	−0.0126 (10)
N2A	0.0144 (11)	0.0293 (14)	0.0281 (13)	−0.0006 (10)	−0.0059 (10)	−0.0130 (11)
C8A	0.0134 (13)	0.0323 (17)	0.0236 (15)	−0.0002 (12)	−0.0037 (11)	−0.0115 (13)
C9A	0.0163 (13)	0.0207 (14)	0.0250 (15)	0.0014 (11)	−0.0053 (11)	−0.0105 (12)
C10A	0.0150 (13)	0.0240 (15)	0.0288 (16)	−0.0027 (11)	−0.0044 (11)	−0.0093 (12)
C11A	0.0210 (14)	0.0206 (15)	0.0265 (15)	−0.0008 (11)	−0.0047 (12)	−0.0048 (12)
Br1A	0.02666 (17)	0.03157 (19)	0.03285 (18)	−0.00522 (13)	−0.00377 (13)	−0.00114 (14)
C12A	0.0247 (15)	0.0252 (16)	0.0261 (15)	0.0027 (12)	−0.0103 (12)	−0.0079 (13)
C13A	0.0159 (14)	0.0317 (17)	0.0311 (16)	0.0014 (12)	−0.0090 (12)	−0.0129 (13)
C14A	0.0159 (13)	0.0232 (15)	0.0266 (15)	−0.0002 (11)	−0.0030 (11)	−0.0118 (12)
O2A	0.0134 (10)	0.0361 (12)	0.0281 (11)	−0.0049 (9)	−0.0044 (8)	−0.0048 (9)
N1B	0.0178 (12)	0.0336 (14)	0.0213 (12)	0.0013 (10)	−0.0078 (10)	−0.0085 (11)
C1B	0.0179 (14)	0.0263 (15)	0.0170 (13)	−0.0048 (11)	−0.0011 (11)	−0.0023 (12)
C2B	0.0284 (16)	0.0340 (18)	0.0206 (15)	−0.0084 (13)	−0.0054 (12)	−0.0077 (13)
C3B	0.0310 (17)	0.0319 (18)	0.0263 (16)	−0.0092 (14)	0.0020 (13)	−0.0129 (14)
C4B	0.0252 (16)	0.0282 (17)	0.0370 (18)	−0.0018 (13)	0.0022 (14)	−0.0144 (14)
C5B	0.0189 (14)	0.0298 (17)	0.0309 (17)	−0.0025 (12)	−0.0052 (12)	−0.0082 (13)
C6B	0.0173 (13)	0.0248 (15)	0.0208 (14)	−0.0040 (11)	−0.0032 (11)	−0.0068 (12)
C7B	0.0165 (13)	0.0271 (16)	0.0240 (15)	−0.0051 (11)	−0.0039 (11)	−0.0071 (12)
O1B	0.0157 (10)	0.0404 (13)	0.0443 (13)	−0.0008 (9)	−0.0090 (9)	−0.0226 (11)
N2B	0.0167 (12)	0.0212 (13)	0.0343 (14)	−0.0003 (10)	−0.0080 (10)	−0.0133 (11)
C8B	0.0153 (13)	0.0279 (16)	0.0246 (15)	0.0021 (11)	−0.0078 (11)	−0.0094 (12)
C9B	0.0177 (13)	0.0233 (15)	0.0240 (15)	0.0020 (11)	−0.0070 (11)	−0.0117 (12)
C10B	0.0200 (14)	0.0252 (15)	0.0243 (15)	0.0030 (12)	−0.0069 (12)	−0.0133 (12)
C11B	0.0291 (16)	0.0263 (16)	0.0217 (15)	0.0064 (13)	−0.0096 (12)	−0.0108 (13)
Br1B	0.0462 (2)	0.0396 (2)	0.02454 (17)	0.00589 (16)	−0.01352 (15)	−0.00780 (14)
C12B	0.0254 (16)	0.0275 (17)	0.0284 (16)	−0.0011 (13)	0.0008 (13)	−0.0080 (13)
C13B	0.0167 (14)	0.0287 (16)	0.0337 (17)	0.0006 (12)	−0.0054 (12)	−0.0119 (14)
C14B	0.0186 (14)	0.0220 (15)	0.0247 (15)	0.0044 (11)	−0.0069 (12)	−0.0104 (12)
O2B	0.0153 (10)	0.0376 (13)	0.0288 (11)	0.0000 (9)	−0.0095 (8)	−0.0049 (10)

### Geometric parameters (Å, °)

**Table:**

N1A—C1A	1.384 (4)	N1B—C1B	1.379 (4)
N1A—C8A	1.453 (4)	N1B—C8B	1.457 (4)
N1A—H1NA	0.8822	N1B—H1NB	0.9273
C1A—C2A	1.402 (4)	C1B—C6B	1.397 (4)
C1A—C6A	1.405 (4)	C1B—C2B	1.408 (4)
C2A—C3A	1.373 (4)	C2B—C3B	1.380 (4)
C2A—H2A	0.9500	C2B—H2B	0.9500
C3A—C4A	1.393 (5)	C3B—C4B	1.389 (5)
C3A—H3A	0.9500	C3B—H3B	0.9500
C4A—C5A	1.375 (4)	C4B—C5B	1.377 (4)
C4A—H4A	0.9500	C4B—H4B	0.9500
C5A—C6A	1.402 (4)	C5B—C6B	1.396 (4)
C5A—H5A	0.9500	C5B—H5B	0.9500
C6A—C7A	1.474 (4)	C6B—C7B	1.463 (4)
C7A—O1A	1.242 (3)	C7B—O1B	1.252 (3)
C7A—N2A	1.342 (4)	C7B—N2B	1.340 (4)
N2A—C8A	1.459 (3)	N2B—C8B	1.461 (3)
N2A—H2NA	0.9523	N2B—H2NB	0.9095
C8A—C9A	1.528 (4)	C8B—C9B	1.522 (4)
C8A—H8A	1.0000	C8B—H8B	1.0000
C9A—C10A	1.388 (4)	C9B—C10B	1.390 (4)
C9A—C14A	1.405 (4)	C9B—C14B	1.399 (4)
C10A—C11A	1.387 (4)	C10B—C11B	1.382 (4)
C10A—H10A	0.9500	C10B—H10B	0.9500
C11A—C12A	1.381 (4)	C11B—C12B	1.377 (4)
C11A—Br1A	1.903 (3)	C11B—Br1B	1.894 (3)
C12A—C13A	1.386 (4)	C12B—C13B	1.385 (4)
C12A—H12A	0.9500	C12B—H12B	0.9500
C13A—C14A	1.386 (4)	C13B—C14B	1.391 (4)
C13A—H13A	0.9500	C13B—H13B	0.9500
C14A—O2A	1.368 (3)	C14B—O2B	1.370 (3)
O2A—H2OA	0.8493	O2B—H2OB	0.8511
			
C1A—N1A—C8A	116.9 (2)	C1B—N1B—C8B	117.7 (2)
C1A—N1A—H1NA	115.4	C1B—N1B—H1NB	113.3
C8A—N1A—H1NA	115.0	C8B—N1B—H1NB	121.5
N1A—C1A—C2A	122.7 (3)	N1B—C1B—C6B	119.3 (3)
N1A—C1A—C6A	118.9 (3)	N1B—C1B—C2B	121.6 (3)
C2A—C1A—C6A	118.3 (3)	C6B—C1B—C2B	119.0 (3)
C3A—C2A—C1A	120.2 (3)	C3B—C2B—C1B	119.8 (3)
C3A—C2A—H2A	119.9	C3B—C2B—H2B	120.1
C1A—C2A—H2A	119.9	C1B—C2B—H2B	120.1
C2A—C3A—C4A	121.3 (3)	C2B—C3B—C4B	121.0 (3)
C2A—C3A—H3A	119.3	C2B—C3B—H3B	119.5
C4A—C3A—H3A	119.3	C4B—C3B—H3B	119.5
C5A—C4A—C3A	119.4 (3)	C5B—C4B—C3B	119.6 (3)
C5A—C4A—H4A	120.3	C5B—C4B—H4B	120.2
C3A—C4A—H4A	120.3	C3B—C4B—H4B	120.2
C4A—C5A—C6A	120.0 (3)	C4B—C5B—C6B	120.5 (3)
C4A—C5A—H5A	120.0	C4B—C5B—H5B	119.8
C6A—C5A—H5A	120.0	C6B—C5B—H5B	119.8
C5A—C6A—C1A	120.5 (3)	C5B—C6B—C1B	120.1 (3)
C5A—C6A—C7A	120.5 (3)	C5B—C6B—C7B	121.3 (3)
C1A—C6A—C7A	118.7 (3)	C1B—C6B—C7B	118.5 (3)
O1A—C7A—N2A	120.9 (3)	O1B—C7B—N2B	120.1 (3)
O1A—C7A—C6A	123.0 (3)	O1B—C7B—C6B	122.8 (3)
N2A—C7A—C6A	115.9 (2)	N2B—C7B—C6B	117.0 (2)
C7A—N2A—C8A	122.3 (2)	C7B—N2B—C8B	122.6 (2)
C7A—N2A—H2NA	114.5	C7B—N2B—H2NB	117.5
C8A—N2A—H2NA	122.0	C8B—N2B—H2NB	119.5
N1A—C8A—N2A	107.8 (2)	N1B—C8B—N2B	107.5 (2)
N1A—C8A—C9A	113.3 (2)	N1B—C8B—C9B	113.8 (2)
N2A—C8A—C9A	112.4 (2)	N2B—C8B—C9B	112.8 (2)
N1A—C8A—H8A	107.7	N1B—C8B—H8B	107.5
N2A—C8A—H8A	107.7	N2B—C8B—H8B	107.5
C9A—C8A—H8A	107.7	C9B—C8B—H8B	107.5
C10A—C9A—C14A	118.8 (3)	C10B—C9B—C14B	118.7 (3)
C10A—C9A—C8A	124.0 (2)	C10B—C9B—C8B	122.6 (2)
C14A—C9A—C8A	117.2 (2)	C14B—C9B—C8B	118.6 (2)
C11A—C10A—C9A	119.9 (3)	C11B—C10B—C9B	120.2 (3)
C11A—C10A—H10A	120.0	C11B—C10B—H10B	119.9
C9A—C10A—H10A	120.0	C9B—C10B—H10B	119.9
C12A—C11A—C10A	121.4 (3)	C12B—C11B—C10B	121.2 (3)
C12A—C11A—Br1A	119.1 (2)	C12B—C11B—Br1B	120.0 (2)
C10A—C11A—Br1A	119.4 (2)	C10B—C11B—Br1B	118.7 (2)
C11A—C12A—C13A	119.1 (3)	C11B—C12B—C13B	119.3 (3)
C11A—C12A—H12A	120.5	C11B—C12B—H12B	120.4
C13A—C12A—H12A	120.5	C13B—C12B—H12B	120.4
C14A—C13A—C12A	120.3 (3)	C12B—C13B—C14B	120.2 (3)
C14A—C13A—H13A	119.8	C12B—C13B—H13B	119.9
C12A—C13A—H13A	119.8	C14B—C13B—H13B	119.9
O2A—C14A—C13A	123.3 (2)	O2B—C14B—C13B	122.9 (3)
O2A—C14A—C9A	116.2 (3)	O2B—C14B—C9B	116.7 (3)
C13A—C14A—C9A	120.5 (3)	C13B—C14B—C9B	120.4 (3)
C14A—O2A—H2OA	114.4	C14B—O2B—H2OB	107.1

### Hydrogen-bond geometry (Å, °)

**Table:**

*D*—H···*A*	*D*—H	H···*A*	*D*···*A*	*D*—H···*A*
N2A—H2NA···O1B	0.95	1.97	2.897 (3)	165.2
N2B—H2NB···O1A	0.91	2.05	2.914 (3)	157.3
O2A—H2OA···O1A^i^	0.85	1.90	2.701 (3)	156.7
O2B—H2OB···O1B^ii^	0.85	1.86	2.691 (3)	165.4

Symmetry codes: (i) *x*−1, *y*, *z*; (ii) *x*+1, *y*, *z*.
